# The Toll of Vascular Insufficiency: Implications for the Management of Peripheral Arterial Disease

**DOI:** 10.1155/2016/8249015

**Published:** 2016-02-21

**Authors:** Jun Xu, Ulka Sachdev

**Affiliations:** Division of Vascular Surgery, University of Pittsburgh Medical Center, 200 Lothrop Street, Suite A1011, Pittsburgh, PA 15213, USA

## Abstract

Peripheral artery disease (PAD) can result in limb loss within six months of diagnosis in a subset of patients who cannot undergo endovascular or surgical revascularization yet continues to maintain a marginal position in cardiovascular research. While a body of literature continues to grow describing the role of danger signaling and innate immunity in cardiac biology, the role of these pathways in the ischemic myopathy associated with PAD has not been extensively studied. The following report will review the current literature on the role of Toll-like receptor (TLR) signaling in cardiovascular biology as well as in nonischemic myopathy. While attenuation of TLR signaling has not been shown to be clinically useful in the treatment of infectious inflammation, it may show promise in the management of severe arterial insufficiency.

## 1. Introduction

Peripheral arterial disease (PAD) represents an advanced stage of atherosclerosis that is associated with increased risk of cardiovascular events and can result in clinically significant limb ischemia. In the United States, PAD affects approximately 5 million adults over the age of 40 [1] and is prevalent in more than 20% of patients over the age of 70 [2]. In patients who are not candidates for revascularization with either open surgical or endovascular therapies, limb loss is a significant risk [1]. Amputations in the elderly have been associated with poor functional outcome, particularly in those who are unable to live independently [3, 4]. As a result, preclinical and clinical research in the area of PAD has focused on improving proangiogenic therapy to promote limb salvage, usually by injecting agents directly into ischemic muscle. These studies have included injection of AdVEGF121, an adenovirus encoding a 121-amino-acid sequence of VEGF [5], fibroblast growth factor (FGF) [6], plasmid containing recombinant hepatocyte growth factor (HGF) [7], and more recently cell-based therapies that are harvested from the patient's own bone marrow [8]. Results have been mixed, as some have been performed in the setting of intermittent claudication, and others were performed for critical limb ischemia and tissue loss [5, 8, 9]. Single agent therapy has not been shown to improve wound healing or prevent amputation [5, 7]. While therapies using autogenous tissue repair cells from the bone marrow appear promising in the 6-month interim analysis, the process of harvesting cells and reinjecting them is intensive and requires anesthesia [8]. Furthermore, the mechanisms that drive the short-term success of cell-based therapy are not known. Thus, a novel nonsurgical approach to PAD to improve muscle recovery and angiogenesis in the setting of limb ischemia would be very useful clinically.

The role of innate immunity in promoting angiogenesis and skeletal muscle recovery after ischemic injury and the ways in which it may be modulated to improve outcomes in PAD have not been extensively studied. The innate immune system, including Toll-like receptors (TLRs), is likely to play an important role in angiogenesis in response to limb ischemia from arterial disease. After ischemic injury, release of damage-associated molecular patterns (DAMPs) such as the nuclear protein high mobility group box 1 (HMGB1) from necrotic muscle and endothelial cells alerts the immune system via pattern recognition receptors like TLRs [10]. TLRs mediate inflammatory responses to both endogenous and exogenous pathogens [11], and inflammation is a critical component of neovascularization and tissue repair [12]. For example, arteriogenesis involves the recruitment of inflammatory cells that respond to endothelial cell activation when shear stress is elevated, resulting in the maturation of preexisting, thin-walled collaterals [13]. Similarly, angiogenesis is supported by macrophages that infiltrate into wounded or ischemic tissue and secrete factors such as VEGF [14]. Studies that have investigated the role of TLR4 signaling in neovascularization have found it to mediate critical events such as arteriogenesis and HMGB-1 mediated angiogenesis in animal models [15, 16].

A number of papers have described potential roles for TLR signaling in the pathophysiology of cardiovascular disease, specifically addressing ways in which TLR activity can modulate systemic inflammation after ischemia, development of atherosclerotic plaques, and modulation of cardiac function following ischemic injury [17–22]. Other lines of research have focused on ways in which TLR signaling contributes to muscle development and pathology in the lower extremity, distinct from cardiac physiology [23]. However, there is a paucity of research describing a potential role for TLR function, danger signaling, and innate immunity in the etiology of ischemic myopathy associated with peripheral arterial disease. The following report will present a review of innate immunity and danger signaling, focusing on the relevance in cardiac disease, myopathy, and ischemia-induced angiogenesis. The purpose of this report is to lay the groundwork for more research into how modulation of TLR signaling may be clinically useful in PAD.

## 2. Review of Innate Immunity

Janeway hypothesized that, during pathogen invasion, the host is able to mount an immediate response via preexisting receptors [24]. Pattern recognition receptors (PRRs) detect conserved components of microorganisms, which signify the presence of microbial nonself. The downstream reactions initiate a cascade of cellular events to eliminate the invading pathogens, making up the earliest stages of an immune response [24]. PRRs detect and bind both pathogen-associated molecular patterns (PAMPs) and damage-associated molecular patterns (DAMPs). PAMPs are small molecules of microbial components such as bacterial lipopolysaccharide (LPS), a constitutive component of the outer cell membrane of Gram-negative bacteria [25]. Other examples of PAMPs include bacterial flagellin, a protein that forms the bacterial flagellum, peptidoglycans, a polymer layer of amino acids and sugar that forms the bacterial cell, glucans on Gram-negative bacteria, and bacterial DNA and RNA [26–28].

The evolutionarily conserved nature of PAMPs allows the innate immune system to quickly recruit immune cells, such as macrophages and leukocytes, remove foreign substances, and initiate processes to protect the host from infection.

In addition to PAMPs, DAMPs are a group of tissue or cell-derived molecules that are released following tissue injury that can activate the innate immune system through PRR. Immune responses are therefore initiated by tissue damage, trauma, and ischemia and are independent of the presence of invasive pathogens. HMGB1 is a nuclear protein and DAMP which functions in this way, promoting systemic inflammation following hemorrhage and hepatic ischemia-reperfusion injury [10, 29, 30]. It can be actively or passively released during injury and ischemia to modulate local and systemic effects [10]. DAMPs such as those derived by degradation of extracellular matrix may also signal the body to repair and replace damaged tissue [31]. Heat-shock proteins (HSP) are DAMPs released by cells when they are exposed to stress such as UV light, heat, and cold [32]. Clinically, DAMPs have been linked to inflammatory diseases such as atherosclerosis, sepsis, and arthritis [33–36]. While DAMPs may contribute to the etiology of disease, some signals may be reparative which is a particularly important concept in the development of novel therapeutics.

The Toll-like receptors (TLRs) are a class of PRRs known to recognize a number of bacterial and viral components, as well as endogenous danger signals [19, 37]. TLR4 is the principal receptor for LPS, working with coreceptors such as CD14 and MD2 to enhance reactivity [38, 39]. While TLR4 plays an important role in LPS recognition as a PRR, it lacks LPS-binding activity without MD2 [40]. Upon recognition of LPS, TLR4 stimulates downstream signaling pathways mediated by myeloid differentiation primary response gene 88 (MyD88) and TIR-domain-containing adaptor-inducing interferon *β* (TRIF). MyD88 signals through interleukin-1 receptor-associated kinase (IRAK), an enzyme that results in translocation of NF*κ*B to the nucleus in a rapid fashion. TRIF also can result in NF*κ*B translocation but with slower kinetics [11]. TLR2 is a PRR that recognizes bacterial lipoproteins such as lipoteichoic acid [19]. Both TLR4 and TLR2 have been shown to play a role in mediating repair and recovery in cardiovascular disease, and both also recognize the danger signal HMGB1 [41].

## 3. TLR Signaling in Atherosclerosis

The role of Toll-like receptor signaling in the etiology of atherosclerosis as well as in cardiomyocyte function following ischemic injury has been well documented [17, 42–45]. A review of these studies can be found in Table 1. In vitro, TLR4 has been detected in endothelial cells and has also been shown to upregulate the expression of TLR2 in both arterial and pulmonary ECs [46, 47]. Those studies have typically demonstrated upregulation of TLR4 (and TLR2) by hypoxia or some other inflammatory insult. In vivo, however, TLR4 expression in normal human arteries such as the superficial temporal artery has been shown to be quite minimal on the endothelial surface, existing only in dendritic cells near the adventitial border [48]. In that same study, both TLR4 and TLR5 were found to be differentially expressed spatially within the arterial wall, stimulating different types of immune activation and vasculitis when stimulated with either LPS (a TLR4 agonist) or flagellin (a TLR5 agonist) [48]. While TLR4 expression in normal arterial segments may be diminutive, expression in atherosclerotic plaques has consistently been found to be elevated both in endothelial cells and in macrophages of endarterectomy specimens [22, 49–51]. Because of its elevated presence in these lesions, a role has been proposed for TLR4 in atherosclerotic plaque formation. A known human TLR4 polymorphism (*TLR4 Asp299Gly*) in which responsiveness to TLR4 ligands is diminished has been studied quite rigorously as possibly being associated with an attenuated risk of cardiovascular diseases [42, 52, 53]. One study evaluated 15 case-control studies to better define the association between* TLR4 Asp299Gly* and atherosclerotic risk, with the premise that polymorphisms may be protective against disease. The investigators found no definitive link between the polymorphism and attenuated atherosclerosis [53]. Thus, an increase in TLR4 expression within plaque is likely a reactive finding rather than an etiologic target.

Once present on a pathologic arterial lesion, TLR4 may mediate plaque activation and rupture [22]. In one study published in 2012, microvascular endothelial cells were found to be significantly more responsive to LPS than macrovascular endothelial cells. The authors suggested that microvascular endothelial cell activation by TLR4 agonists might promote plaque destabilization, a critical event in acute cardiovascular syndromes [50]. This group also identified that microvascular endothelial cells contained higher levels of CD14, an important coreceptor for TLR4 that can enhance reactivity. Levels of MD2, another important coreceptor, did not differ between the cell types in that study [50]. Recently, Yang et al. have demonstrated that MD2 is critical for HMGB1-TLR4 interactions as well as HMGB1-mediated inflammatory responses in vivo. These experiments were performed in vitro using macrophages and monocytes, as well as in vivo in models of hepatic injury [54]. However, one hypothesis is that MD2 is likely to be important for HMGB-1-TLR4 interactions in endothelial cells as well.

An important etiologic factor in the development of atherosclerosis is the incorporation of lipids into inflammatory cells and intimal lesions. TLR4 has been demonstrated to mediate uptake of minimally oxidized LDL (mmLDL) in circulation by monocytes, as well as in tissue by activated macrophages [55]. Cytoskeletal rearrangements in macrophages exposed to mmLDL appear to be a response to bioactive cholesteryl esters within the molecule. Macropinocytosis, or uptake of small particles within fluid that is associated with the ruffled appearance of activated macrophages, occurred in TLR4-competent cells exposed to mmLDL. Interestingly, this phenomenon was largely a MyD88-independent event, perhaps signifying an important role for TRIF-mediated signaling downstream of TLR4 in these processes [55].

Other TLRs have been posited to play a role in atherosclerotic lesion formation. Citing inflammation as an initial insult for plaque development, Zimmer et al. investigated the role of TLR3, a receptor for viral double stranded RNA that signals through TRIF [56]. TLR3 can also respond to endogenous ligands released locally from tissue damage. In this paper, control mice, or those with deficient TLR3 signaling, were injected with the double stranded RNA analog polyinosinic polycytidylic acid (Poly:IC). Wild-type mice injected with Poly:IC developed impaired endothelium-dependent vasodilation and development of reactive oxygen species from aortic rings. TLR3 knockout mice maintained normal vasodilatation and attenuated reactive oxygen species with Poly:IC treatment. Reendothelialization after denudation injury was also impaired in WT mice exposed to Poly:IC but maintained in TLR3 knockout mice. The authors of this report suggested that TLR3 activation by endogenous ligands released at the time of arterial injury may be an etiologic factor of all stages of atherosclerosis, given its constitutive expression on EC, and the potential for injury in surrounding areas [56].

## 4. TLR Signaling in Cardiomyocyte Function

Toll-like receptors like TLR4 expression have also been demonstrated on cardiomyocytes. In 1999, Frantz et al. demonstrated TLR4 expression in both normal and failing myocardium [17]. In that early report, TLR4 expression was found to be present not only in cardiomyocytes but also in microvascular cells within the hearts of both rodents and humans. Additionally, the presence of congestive heart failure resulted in focal, intense staining of TLR4 [17]. One of the prevailing theories regarding the role of TLR signaling in the heart during sterile conditions was its ability to mediate engulfment of apoptotic cells. Since that time, a number of studies have postulated a role for TLR4 in cardiomyocyte apoptosis as well as cardiac muscle remodeling after injury [45, 57]. Interestingly, LPS, the main ligand for TLR4, has been shown to be antiapoptotic and protective in the heart. Administration of LPS to cardiomyocytes in culture as well as in vivo resulted in improved contractility as well as diminished apoptosis in a MyD88 and NOS2 dependent manner [45].

In contrast, activation of TLR4 through endogenous danger signals such as HSP60 has been shown to be proapoptotic and attenuated using antagonists of TLR4 activity [57]. Administration of HSP60 resulted in greater expression of activated caspase-3 as well as increased DNA fragmentation in cardiac myocytes. Heat inactivated HSP60 as well as administration of TLR4 neutralizing antibodies attenuated this effect. Antibodies against TLR2 and CD14 had no effect on apoptosis under similar conditions [57]. In another study by Timmer et al., functional TLR4 was associated with negative left ventricular remodeling and function following myocardial infection. In those experiments, investigators utilized C3H/HeJ mice, which exhibit TLR4 incompetence rather than absence [58]. TLR4 incompetence was associated with preservation of systolic function, diminished interstitial fibrosis, and attenuated myocardial hypertrophy. These findings were similarly associated with diminished expression of inflammatory cytokines, tumor necrosis factor alpha, interferon-gamma, granulocyte/macrophage-colony-stimulating factor, and matrix metalloproteinase-2 in C3H/HeJ mice compared to controls [58]. These data suggest that attenuating TLR4 activity in muscle ischemia may be an important therapeutic intervention in the management of ischemic cardiomyopathy and congestive heart failure.

TLR2 has also been shown to play a role in apoptotic pathways within cardiac muscle. In one study by Frantz et al., neutralizing antibodies to TLR2 enhanced oxidative stress in rat ventricular myocytes [59]. Hydrogen peroxide- (H_2_O_2_-) induced cytotoxicity was promoted in cells treated with TLR2 antagonism, suggested by higher levels of annexin-staining cells. TLR2 also was shown to result in NF*κ*B translocation in hamster ovary cells in response to H_2_O_2_, suggesting that reactive oxygen species may be a target of TLR2 signaling. In this study, activation of TLR2 was found to be protective in cardiac myocytes [59]. Others have also demonstrated a protective, proangiogenic role for TLR2 in response to endogenous oxidative signals [60].

## 5. TLR Signaling in Nonischemic Myopathy

A role for Toll-like receptor activity has been described in the etiology of myopathy and myositis, distinct from ischemic insults. In 2006, Schreiner et al. evaluated expression of TLRs 1–9 on myoblasts as well as human rhabdomyosarcoma cells. They demonstrated that muscle cells demonstrated low levels of TLRs 1–7 as well as TLR9 by analysis of protein as well as mRNA levels. Interestingly, expression of TLR3 could be upregulated with activation by its own ligand Poly:IC as well as interferon-gamma. Not surprisingly, the expression of TLR3 was largely intracellular, as it is an endosomal receptor for double stranded RNA [61]. Activation of TLR3 resulted in translocation of NF-*κ*B and release of interleukin-8 in cultured cells. Finally, these researchers demonstrated high levels of TLR3 in inclusion bodies found within myocytes of patients with HIV induced inclusion-body myositis [61].

In one recent study by Zong et al., TLR4 was found to be expressed in the myoplasm of skeletal muscle biopsies taken from both healthy subjects as well as those from polymyositis (PM) and dermatomyositis (DM) patients. While the amount of TLR4 in patients and control subjects was not different, colocalization with major histocompatibility complex-1 (MHC-1) was seen only in PM and DM patients [62]. Furthermore, in mice, HMGB1 was found to increase the expression of MHC-1 on flexor digitorum brevis (FDB) fibers in a TLR4-dependent manner. The redox state of HMGB1 has been shown by others to be critical to its functionality as well as its localization within the cell [63]. Zong et al. further specified that maintenance of a thiol group in cysteine (C) 106 and a disulfide bond between C23 and C45 on the HMGB1 molecule was required for interaction between HMGB1 and TLR4 on FDB muscle fibers [62].

In another study evaluating mouse models of muscular dystrophy, TLR7 appeared to play an important role in inflammatory responses in dystrophin-deficient muscle cells. Specifically, myoblasts harvested from the diaphragm, gastrocnemius, and soleus of dystrophic mice (mdx) expressed a number of Toll-like receptors including TLR2, TLR4, TLR7, and TLR9. Additionally, myoblasts produced cytokines when activated by specific TLR ligands, including single stranded RNA (ssRNA), an activator of TLR7 signaling. When mdx mice were bred with MyD88 knockout mice, they demonstrated improved hindlimb strength, suggesting that this critical mediator of most TLR signaling contributes to the pathophysiology of weakness in dystrophic mice [64].

## 6. Danger Signaling in Ischemia-Induced Angiogenesis

TLR4 antagonism has not been proposed as a therapy for the management of ischemic muscle injury and its clinical sequelae. Furthermore, the majority of studies have investigated TLR function in the setting of cardiomyocyte ischemia and not the ischemia that affects skeletal muscle induced by peripheral arterial disease. For this reason, our laboratory has been invested in studying how danger signaling and TLR activity modulates responses to skeletal muscle ischemia, using mouse models of induced myocardial injury. If modulation of TLR signaling improves skeletal muscle function in the setting of ischemia, many patients with significant peripheral arterial disease who are not candidates for revascularization, or who are impaired by intermittent claudication, may benefit from this novel line of therapy.

The innate immune system is likely to play an important role in angiogenesis, particularly in response to tissue ischemia. Ischemia in brain, muscle, and liver has been shown to result in release of the danger signal HMGB1 from its normal nuclear stores [65, 66]. Release suggests that HMGB1 can then act locally and/or systemically to mediate both inflammatory and restorative processes. HMGB1 is a highly conserved and ubiquitous nuclear protein that can be secreted by activated macrophages and dendritic cells. It is passively released by necrotic but not apoptotic cells [10, 67–69]. Extracellular HMGB1 functions as a cytokine and, in animal models, has been shown to be a late mediator of lethality in sepsis, as well as a mediator of remote organ damage after tissue injury [29, 30, 67, 70, 71]. Neutralizing antibodies to HMGB1 attenuates liver damage in animal models of ischemia-reperfusion injury, supporting its role in mediating systemic inflammatory effects [29, 30, 71]. In the setting of lower extremity ischemia, hypoxic muscle cell injury is prominent and may serve as a large source of local HMGB1 release. A number of studies report potent effects of HMGB1 on vascular cells. Palumbo et al. have shown that HMGB1 induces the proliferation and migration of mesoangioblasts [72], vessel-associated stem cells that are home to damaged muscle and engage in regeneration [73]. Degryse et al. reported an effect of HMGB1 on chemotaxis and cytoskeletal reorganization in rat smooth muscle cells [74]. Additionally, HMGB1 has been shown to induce migration of endothelial progenitor cells [75], EC sprouting [76], and cell migration in wounded endothelial monolayers [77]. In vivo, HMGB1 has been shown to be critical to axonal elongation in the central nervous system [78–81] and may play an analogous role in the vascular system [82]. Administration of intramuscular HMGB1 in the setting of hindlimb ischemia increased limb perfusion by laser Doppler perfusion imaging (LDPI), led to the development of more mature collaterals than in control treated animals, and increased the number of regenerating muscle fibers [83]. The first recognized receptor for the extracellular effects of HMGB1 was RAGE [84]. Advanced glycation end-products activate RAGE and initiate an inflammatory response. RAGE has been implicated in a variety of diseases including diabetes and neurological disorders [84] and has been shown to mediate axonal regeneration in a HMGB1 dependent fashion [78, 79, 85]. RAGE has been shown to mediate EC sprouting and migration in response to HMGB1 [77] and until recently had been the only receptor linked to a possible role in HMGB1-mediated angiogenesis [77].

TLR4-mediated inflammation has been shown to be important in arteriogenesis, the maturation of preexisting collaterals in the setting of increased shear stress [15]. This would suggest that TLR4 is protective in the setting of tissue ischemia, promoting neovascularization and improved tissue perfusion. However, others, including our laboratory, have demonstrated mixed results regarding the role of TLR4 in response to muscle ischemia. In mice in which TLR4 is present but nonfunctional, loss of TLR4 is detrimental leading to increased tissue necrosis and a poor angiogenic response [66]. Interestingly, antagonism of HMGB1 with antibody results in similar findings [66]. In contrast, C57B6 mice in which TLR4 is genetically absent (TLR4KO) demonstrate faster return of perfusion to a limb rendered ischemic by femoral artery ligation. Loss of the lipoprotein receptor TLR2, however, results in pronounced necrosis, abnormal appearing vessels, and potentially lack of protection against TLR4 mediated inflammation [47, 86]. These findings would suggest that the interplay of TLR4 and other TLRs such as TLR2 is critical in stemming overwhelming inflammation and tissue damage.

**Figure 1:**
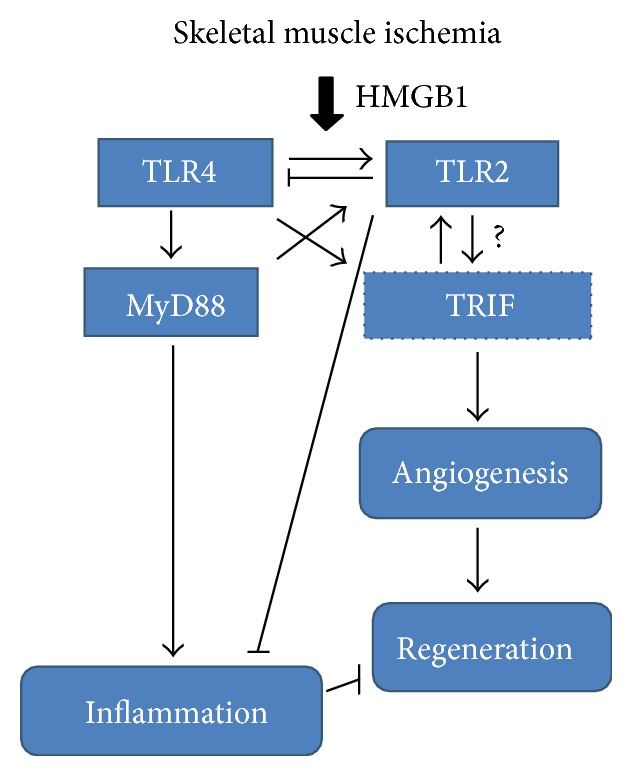
Proposed pathway connecting HMGB1, TLR4, TLR2, MyD88, and TRIF signaling to inflammation and regeneration following muscle ischemia. We have shown that HMGB1 is released from ischemic myocytes and TLRs 2 and 4 have opposing roles following hindlimb ischemia. This proposed mechanism demonstrates the interplay between TLR4, TLR2, MyD88, and TRIF in response to skeletal muscle ischemia.

We have also demonstrated similar interplay between MyD88 and TRIF. Genetic absence of MyD88 results in diminished IL-6 levels after ischemia and attenuated recruitment of inflammatory cells. In contrast, TRIF absence resulted in a prolonged inflammatory state, with IL6 levels remaining elevated up to a week after the initial injury [87]. Furthermore, inflammatory cell recruitment was elevated in the absence of TRIF, and regeneration severely diminished [86, 87]. These results mimicked those of TLR2KO mice; however, TLR2 is not known to signal through TRIF. These findings suggest that, in the setting of limb ischemia, TLR pathways may demonstrate complex and potentially novel interactions to promote both inflammation and recovery from the injury (Figure 1). These pathways remain a significant focus of study in our laboratory.

## 7. TLR4 Modulation in PAD: Potential New Avenue for Investigation

Modulation of innate immunity, danger signaling, and Toll-like receptor activity has not been a major focus in the management of PAD. However, inferences from their roles in cardiac pathology in both human disease and animal models suggest that their pathways may present new therapeutic targets for arterial disease. TLR4 antagonism has been tested in sepsis with disappointing results [38], but it is possible that these agents can be repurposed to help manage the consequences of peripheral ischemia. This avenue of research may be beneficial to pursue to help define a new line of therapy for patients with severe PAD.

A theoretical concern exists that overwhelming immunosuppression may occur by employing antagonists of TLR function to combat disease. However, antagonism of TLR signaling has been proposed and investigated in a variety of infectious disease processes without such consequences and is reviewed by Savva and Roger [88]. Because TLR function is often redundant, inhibition of single TLRs may not necessarily result in overwhelming immunosuppression. By the same argument, targeting of a single TLR may limit effectiveness of therapy. Both hypotheses remain to be tested vigorously. Eritoran tetrasodium (E5564; Eritoran, Eisai; Andover, MA) is a second-generation lipid-A analog that targets LPS-TLR4-MD2 interaction. In preclinical studies, it was found to diminish cytokine release from human monocytes exposed to LPS, including expression of TNF-alpha. Furthermore, it reduced mortality in mice exposed to LPS in a dose-dependent fashion [89]. However, in the ACCESS trial, eritoran had no effect in reducing 28-day patient mortality due to sepsis [38]. Treatment-emergent adverse events including atrial fibrillation, hepatic dysfunction, hemorrhagic events, and phlebitis were similar between treatment and placebo groups [38]. In regard to side effects, when administered to normal volunteers in early phase studies, eritoran was associated with a dose-dependent phlebitis at the injection site [90]. Volunteers were studied for approximately 1-2 weeks following infusion. Other side effects such as dyspepsia, gastrointestinal discomfort, fever, vasodilation, LFT abnormalities, pharyngitis, and conjunctivitis were not different between placebo and eritoran groups [90].

Whether anti-TLR4 treatments would be effective in managing PAD remains to be investigated, but the suggestion represents a novel approach to treating patients with cardiovascular disease. While data from knockout mice suggest that diminishing TLR4 activity may offer promising results in improving both perfusion and muscle recovery in the short term, further studies employing exogenously administered TLR4 antagonists would be required in preclinical experiments. Modulation of TLR activity in cardiovascular biology remains an intriguing line of investigation, particularly as it relates to patients with severe lower extremity arterial insufficiency.

## Figures and Tables

**Table 1:** Role of TLR in cardiovascular biology.

Toll-like receptor (TLR)	Ligands	Cell type	Proposed disease process affected	Role	Reference
TLR2	Carboxy alkyl pyrroles	Endothelial cells	Cancer	Proangiogenic	[60]
	HSP60^a^	Cardiac myocytes		Antiapoptotic	[59]

TLR3	Poly:IC	Skeletal myocytes	HIV myopathy	Proinflammatory	[61]
	Double stranded RNA	Endothelial cells	Atherosclerosis	Proapoptotic	[56]

TLR4	HMGB1	Skeletal myocytes	Myositis	Proinflammatory	[62]
	mmLDL^b^	Macrophages	Atherosclerosis	Increases uptake	[55]
	EDA^c^	Cardiac myocytes	Heart failure	Proinflammatory	[58]
	LPS	Endothelial cells	Plaque rupture	Proinflammatory	[50]
	LPS	Endothelial cells	Panarteritis	Proinflammatory	[48]
	LPS	Cardiac myocytes	Heart failure	Antiapoptotic	[45]
	HSP60	Cardiac myocytes	Heart failure	Proapoptotic	[57]

TLR5	Flagellin	Endothelial cells	Adventitial vasculitis	Proinflammatory	[48]

TLR7	Single stranded RNA	Skeletal myocytes	Muscular dystrophy	Proinflammatory	[64]

Current literature suggests that multiple TLRs play a role in cardiovascular disease. This table describes the TLR, proposed ligand, and cell type that is affected as it relates to disease process involving the vascular system. References are also noted. ^a^HSP60 is suggested by the authors as a possible TLR2 ligand for the observed effect. ^b^Minimally oxidized low density lipoprotein; ^c^alternatively spliced extra domain A of fibronectin.

## References

[1] Selvin E., Erlinger T. P. (2004). Prevalence of and risk factors for peripheral arterial disease in the United States: results from the National Health and Nutrition Examination Survey, 1999-2000. *Circulation*.

[2] Ostchega Y., Paulose-Ram R., Dillon C. F., Gu Q., Hughes J. P. (2007). Prevalence of peripheral arterial disease and risk factors in persons aged 60 and older: data from the National Health and Nutrition Examination Survey 1999–2004. *Journal of the American Geriatrics Society*.

[3] Vogel T. R., Petroski G. F., Kruse R. L. (2014). Impact of amputation level and comorbidities on functional status of nursing home residents after lower extremity amputation. *Journal of Vascular Surgery*.

[4] Vogel T. R., Petroski G. F., Kruse R. L. (2014). Functional status of elderly adults before and after interventions for critical limb ischemia. *Journal of Vascular Surgery*.

[5] Rajagopalan S., Mohler E. R., Lederman R. J. (2003). Regional angiogenesis with vascular endothelial growth factor in peripheral arterial disease: a phase II randomized, double-blind, controlled study of adenoviral delivery of vascular endothelial growth factor 121 in patients with disabling intermittent claudication. *Circulation*.

[6] Lederman R. J., Mendelsohn F. O., Anderson R. D. (2002). Therapeutic angiogenesis with recombinant fibroblast growth factor-2 for intermittent claudication (the TRAFFIC study): a randomised trial. *The Lancet*.

[7] Powell R. J., Simons M., Mendelsohn F. O. (2008). Results of a double-blind, placebo-controlled study to assess the safety of intramuscular injection of hepatocyte growth factor plasmid to improve limb perfusion in patients with critical limb ischemia. *Circulation*.

[8] Powell R. J., Comerota A. J., Berceli S. A. (2011). Interim analysis results from the RESTORE-CLI, a randomized, double-blind multicenter phase II trial comparing expanded autologous bone marrow-derived tissue repair cells and placebo in patients with critical limb ischemia. *Journal of Vascular Surgery*.

[9] Messina L. M., Brevetti L. S., Chang D. S., Paek R., Sarkar R. (2002). Therapeutic angiogenesis for critical limb ischemia: invited commentary. *Journal of Controlled Release*.

[10] Lotze M. T., Tracey K. J. (2005). High-mobility group box 1 protein (HMGB1): nuclear weapon in the immune arsenal. *Nature Reviews Immunology*.

[11] Kawai T., Akira S. (2005). Toll-like receptor downstream signaling. *Arthritis Research and Therapy*.

[12] Frantz S., Vincent K. A., Feron O., Kelly R. A. (2005). Innate immunity and angiogenesis. *Circulation Research*.

[13] Buschmann I., Schaper W. (2000). The pathophysiology of the collateral circulation (arteriogenesis). *Journal of Pathology*.

[14] Jain R. K. (2003). Molecular regulation of vessel maturation. *Nature Medicine*.

[15] Bastiaansen A. J. N. M., Karper J. C., Wezel A. (2014). TLR4 accessory molecule RP105 (CD180) regulates monocyte-driven arteriogenesis in a murine hind limb ischemia model. *PLoS ONE*.

[16] Lin Q., Yang X. P., Fang D. (2011). High-mobility group box-1 mediates toll-like receptor 4-dependent angiogenesis. *Arteriosclerosis, Thrombosis, and Vascular Biology*.

[17] Frantz S., Kobzik L., Kim Y.-D. (1999). Toll4 (TLR4) expression in cardiac myocytes in normal and failing myocardium. *Journal of Clinical Investigation*.

[18] Ma Y., Zhang X., Bao H. (2012). Toll-like receptor (tlr) 2 and tlr4 differentially regulate doxorubicin induced cardiomyopathy in mice. *PLoS ONE*.

[19] Mitchell J. A., Paul-Clark M. J., Clarke G. W., McMaster S. K., Cartwright N. (2007). Critical role of toll-like receptors and nucleotide oligomerisation domain in the regulation of health and disease. *Journal of Endocrinology*.

[20] Riad A., Meyer Zu Schwabedissen H., Weitmann K. (2012). Variants of toll-like receptor 4 predict cardiac recovery in patients with dilated cardiomyopathy. *Journal of Biological Chemistry*.

[21] Vaquero J., Campbell J. S., Haque J. (2011). Toll-like receptor 4 and myeloid differentiation factor 88 provide mechanistic insights into the cause and effects of interleukin-6 activation in mouse liver regeneration. *Hepatology*.

[22] Edfeldt K., Swedenborg J., Hansson G. K., Yan Z.-Q. (2002). Expression of toll-like receptors in human atherosclerotic lesions: a possible pathway for plaque activation. *Circulation*.

[23] Frost R. A., Nystrom G. J., Lang C. H. (2006). Multiple Toll-like receptor ligands induce an IL-6 transcriptional response in skeletal myocytes. *The American Journal of Physiology—Regulatory Integrative and Comparative Physiology*.

[24] Janeway C. A., Medzhitov R. (2002). Innate immune recognition. *Annual Review of Immunology*.

[25] Fitzgerald K. A., Rowe D. C., Barnes B. J. (2003). LPS-TLR4 signaling to IRF-3/7 and NF-*κ*B involves the toll adapters TRAM and TRIF. *Journal of Experimental Medicine*.

[26] Hayashi F., Smith K. D., Ozinsky A. (2001). The innate immune response to bacterial flagellin is mediated by Toll-like receptor 5. *Nature*.

[27] Gust A. A., Biswas R., Lenz H. D. (2007). Bacteria-derived peptidoglycans constitute pathogen-associated molecular patterns triggering innate immunity in *Arabidopsis*. *The Journal of Biological Chemistry*.

[28] Muta T. (2006). Molecular basis for invertebrate innate immune recognition of (1 → 3)-*β*-D-glucan as a pathogen-associated molecular pattern. *Current Pharmaceutical Design*.

[29] Levy R. M., Mollen K. P., Prince J. M. (2007). Systemic inflammation and remote organ injury following trauma require HMGB1. *American Journal of Physiology—Regulatory Integrative and Comparative Physiology*.

[30] Mollen K. P., Levy R. M., Prince J. M. (2008). Systemic inflammation and end organ damage following trauma involves functional TLR4 signaling in both bone marrow-derived cells and parenchymal cells. *Journal of Leukocyte Biology*.

[31] Schaefer L. (2014). Complexity of danger: the diverse nature of damage-associated molecular patterns. *The Journal of Biological Chemistry*.

[32] El Mezayen R., El Gazzar M., Seeds M. C., McCall C. E., Dreskin S. C., Nicolls M. R. (2007). Endogenous signals released from necrotic cells augment inflammatory responses to bacterial endotoxin. *Immunology Letters*.

[33] Schett G., Redlich K., Xu Q. (1998). Enhanced expression of heat shock protein 70 (hsp70) and heat shock factor 1 (HSF1) activation in rheumatoid arthritis synovial tissue: differential regulation of hsp70 expression and HSF1 activation in synovial fibroblasts by proinflammatory cytokines, shear stress, and antiinflammatory drugs. *The Journal of Clinical Investigation*.

[34] Ulloa L., Ochani M., Yang H. (2002). Ethyl pyruvate prevents lethality in mice with established lethal sepsis and systemic inflammation. *Proceedings of the National Academy of Sciences of the United States of America*.

[35] Yang H., Ochani M., Li J. (2004). Reversing established sepsis with antagonists of endogenous high-mobility group box 1. *Proceedings of the National Academy of Sciences of the United States of America*.

[36] Hartvigsen K., Chou M.-Y., Hansen L. F. (2009). The role of innate immunity in atherogenesis. *Journal of Lipid Research*.

[37] Lien E., Sellati T. J., Yoshimura A. (1999). Toll-like receptor 2 functions as a pattern recognition receptor for diverse bacterial products. *The Journal of Biological Chemistry*.

[38] Opal S. M., Laterre P.-F., Francois B. (2013). Effect of eritoran, an antagonist of MD2-TLR4, on mortality in patients with severe sepsis: The ACCESS randomized trial. *The Journal of the American Medical Association*.

[39] Hoshino K., Takeuchi O., Kawai T. (1999). Toll-like receptor 4 (TLR4)-deficient mice are hyporesponsive to lipopolysaccharide: evidence for TLR4 as the Lps gene product. *Journal of Immunology*.

[40] Park H. S., Jung H. Y., Park E. Y., Kim J., Lee W. J., Bae Y. S. (2004). Cutting edge: direct interaction of TLR4 with NAD(P)H oxidase 4 isozyme is essential for lipopolysaccharide-induced production of reactive oxygen species and activation of NF-*κ*B. *The Journal of Immunology*.

[41] Bae J.-S., Rezaie A. R. (2011). Activated protein C inhibits high mobility group box 1 signaling in endothelial cells. *Blood*.

[42] Boekholdt S. M., Agema W. R. P., Peters R. J. G. (2003). Variants of toll-like receptor 4 modify the efficacy of statin therapy and the risk of cardiovascular events. *Circulation*.

[43] Dasu M. R., Devaraj S., Park S., Jialal I. (2010). Increased Toll-Like Receptor (TLR) activation and TLR ligands in recently diagnosed type 2 diabetic subjects. *Diabetes Care*.

[44] Labrum R., Bevan S., Sitzer M., Lorenz M., Markus H. S. (2007). Toll receptor polymorphisms and carotid artery intima-media thickness. *Stroke*.

[45] Zhu X., Zhao H., Graveline A. R. (2006). MyD88 and NOS2 are essential for Toll-like receptor 4-mediated survival effect in cardiomyocytes. *American Journal of Physiology—Heart and Circulatory Physiology*.

[46] Fan J., Frey R. S., Malik A. B. (2003). TLR4 signaling induces TLR2 expression in endothelial cells via neutrophil NADPH oxidase. *The Journal of Clinical Investigation*.

[47] Xu J., Benabou K., Cui X. (2015). TLR4 deters perfusion recovery and upregulates TLR2 in ischemic skeletal muscle and endothelial cells. *Molecular Medicine*.

[48] Deng J., Ma-Krupa W., Gewirtz A. T., Younge B. R., Goronzy J. J., Weyand C. M. (2009). Toll-like receptors 4 and 5 induce distinct types of vasculitis. *Circulation Research*.

[49] Hodgkinson C. P., Laxton R. C., Patel K., Ye S. (2008). Advanced glycation end-product of low density lipoprotein activates the toll-like 4 receptor pathway implications for diabetic atherosclerosis. *Arteriosclerosis, Thrombosis, and Vascular Biology*.

[50] Lu Z., Li Y., Jin J., Zhang X., Lopes-Virella M. F., Huang Y. (2012). Toll-like receptor 4 activation in microvascular endothelial cells triggers a robust inflammatory response and cross talk with mononuclear cells via interleukin-6. *Arteriosclerosis, Thrombosis, and Vascular Biology*.

[51] Miller Y. I., Choi S.-H., Fang L., Harkewicz R. (2009). Toll-like receptor-4 and lipoprotein accumulation in macrophages. *Trends in Cardiovascular Medicine*.

[52] Yang I. A., Holloway J. W., Ye S. (2003). TLR4 Asp299Gly polymorphism is not associated with coronary artery stenosis. *Atherosclerosis*.

[53] Zhang K., Zhang L., Zhou B. (2012). Lack of association between *TLR4 Asp299Gly* polymorphism and atherosclerosis: evidence from meta-analysis. *Thrombosis Research*.

[54] Yang H., Wang H., Ju Z. (2015). MD-2 is required for disulfide HMGB1-dependent TLR4 signaling. *Journal of Experimental Medicine*.

[55] Choi S.-H., Harkewicz R., Lee J. H. (2009). Lipoprotein accumulation in macrophages via toll-like receptor-4-dependent fluid phase uptake. *Circulation Research*.

[56] Zimmer S., Steinmetz M., Asdonk T. (2011). Activation of endothelial toll-like receptor 3 impairs endothelial function. *Circulation Research*.

[57] Kim S.-C., Stice J. P., Chen L. (2009). Extracellular heat shock protein 60, cardiac myocytes, and apoptosis. *Circulation Research*.

[58] Timmers L., Sluijter J. P. G., van Keulen J. K. (2008). Toll-like receptor 4 mediates maladaptive left ventricular remodeling and impairs cardiac function after myocardial infarction. *Circulation Research*.

[59] Frantz S., Kelly R. A., Bourcier T. (2001). Role of TLR-2 in the activation of nuclear factor *κ*B by oxidative stress in cardiac myocytes. *The Journal of Biological Chemistry*.

[60] West X. Z., Malinin N. L., Merkulova A. A. (2010). Oxidative stress induces angiogenesis by activating TLR2 with novel endogenous ligands. *Nature*.

[61] Schreiner B., Voss J., Wischhusen J. (2006). Expression of toll-like receptors by human muscle cells in vitro and in vivo: TLR3 is highly expressed in inflammatory and HIV myopathies, mediates IL-8 release and up-regulation of NKG2D-ligands. *The FASEB Journal*.

[62] Zong M., Bruton J. D., Grundtman C. (2013). TLR4 as receptor for HMGB1 induced muscle dysfunction in myositis. *Annals of the Rheumatic Diseases*.

[63] Tang D., Kang R., Livesey K. M. (2010). Endogenous HMGB1 regulates autophagy. *Journal of Cell Biology*.

[64] Henriques-Pons A., Yu Q., Rayavarapu S. (2014). Role of Toll-like receptors in the pathogenesis of dystrophin-deficient skeletal and heart muscle. *Human Molecular Genetics*.

[65] Qiu J., Nishimura M., Wang Y. (2008). Early release of HMGB-1 from neurons after the onset of brain ischemia. *Journal of Cerebral Blood Flow and Metabolism*.

[66] Sachdev U., Cui X., Tzeng E. (2013). HMGB1 and TLR4 mediate skeletal muscle recovery in a murine model of hindlimb ischemia. *Journal of Vascular Surgery*.

[67] Yang H., Wang H., Czura C. J., Tracey K. J. (2005). The cytokine activity of HMGB1. *Journal of Leukocyte Biology*.

[68] Dumitriu I. E., Baruah P., Manfredi A. A., Bianchi M. E., Rovere-Querini P. (2005). HMGB1: guiding immunity from within. *Trends in Immunology*.

[69] Bianchi M. E. (2009). HMGB1 loves company. *Journal of Leukocyte Biology*.

[70] Wang H., Bloom O., Zhang M. (1999). HMG-1 as a late mediator of endotoxin lethality in mice. *Science*.

[71] Tsung A., Sahai R., Tanaka H. (2005). The nuclear factor HMGB1 mediates hepatic injury after murine liver ischemia-reperfusion. *Journal of Experimental Medicine*.

[72] Palumbo R., Sampaolesi M., De Marchis F. (2004). Extracellular HMGB1, a signal of tissue damage, induces mesoangioblast migration and proliferation. *Journal of Cell Biology*.

[73] Sampaolesi M., Torrente Y., Innocenzi A. (2003). Cell therapy of *α*-sarcoglycan null dystrophic mice through intra-arterial delivery of mesoangioblasts. *Science*.

[74] Degryse B., Bonaldi T., Scaffidi P. (2001). The high mobility group (HMG) boxes of the nuclear protein HMG1 induce chemotaxis and cytoskeleton reorganization in rat smooth muscle cells. *Journal of Cell Biology*.

[75] Chavakis E., Hain A., Vinci M. (2007). High-mobility group box 1 activates integrin-dependent homing of endothelial progenitor cells. *Circulation Research*.

[76] Schlueter C., Weber H., Meyer B. (2005). Angiogenetic signaling through hypoxia: HMGB1: an angiogenetic switch molecule. *The American Journal of Pathology*.

[77] Mitola S., Belleri M., Urbinati C. (2006). Cutting edge: extracellular high mobility group box-1 protein is a proangiogenic cytokine. *The Journal of Immunology*.

[78] Huttunen H. J., Kuja-Panula J., Sorci G., Agneletti A. L., Donato R., Rauvala H. (2000). Coregulation of neurite outgrowth and cell survival by amphoterin and S100 proteins through receptor for advanced glycation end products (RAGE) activation. *The Journal of Biological Chemistry*.

[79] Rauvala H., Pihlaskari R. (1987). Isolation and some characteristics of an adhesive factor of brain that enhances neurite outgrowth in central neurons. *The Journal of Biological Chemistry*.

[80] Rong L. L., Yan S.-F., Wendt T. (2004). RAGE modulates peripheral nerve regeneration via recruitment of both inflammatory and axonal outgrowth pathways. *The FASEB Journal*.

[81] Ling L. R., Trojaborg W., Qu W. (2004). Antagonism of RAGE suppresses peripheral nerve regeneration. *The FASEB Journal*.

[82] Wake H., Mori S., Liu K., Takahashi H. K., Nishibori M. (2009). Histidine-rich glycoprotein inhibited high mobility group box 1 in complex with heparin-induced angiogenesis in matrigel plug assay. *European Journal of Pharmacology*.

[83] De Mori R., Straino S., Di Carlo A. (2007). Multiple effects of high mobility group box protein 1 in skeletal muscle regeneration. *Arteriosclerosis, Thrombosis, and Vascular Biology*.

[84] Herold K., Moser B., Chen Y. (2007). Receptor for advanced glycation end products (RAGE) in a dash to the rescue: inflammatory signals gone awry in the primal response to stress. *Journal of Leukocyte Biology*.

[85] Andrassy M., Volz H. C., Igwe J. C. (2008). High-mobility group box-1 in ischemia-reperfusion injury of the heart. *Circulation*.

[86] Sachdev U., Cui X., McEnaney R., Wang T., Benabou K., Tzeng E. (2012). TLR2 and TLR4 mediate differential responses to limb ischemia through MyD88-dependent and independent pathways. *PLoS ONE*.

[87] Sachdev U., Cui X., Xu J., Xu J., Tzeng E. (2014). MyD88 and TRIF mediate divergent inflammatory and regenerative responses to skeletal muscle ischemia. *Physiological Reports*.

[88] Savva A., Roger T. (2013). Targeting Toll-like receptors: promising therapeutic strategies for the management of sepsis-associated pathology and infectious diseases. *Frontiers in Immunology*.

[89] Mullarkey M., Rose J. R., Bristol J. (2003). Inhibition of endotoxin response by E5564, a novel toll-like receptor 4-directed endotoxin antagonist. *Journal of Pharmacology and Experimental Therapeutics*.

[90] Rossignol D. P., Wong N., Noveck R., Lynn M. (2008). Continuous pharmacodynamic activity of eritoran tetrasodium, a TLR4 antagonist, during intermittent intravenous infusion into normal volunteers. *Innate Immunity*.

